# Whole transcriptome screening for novel genes involved in meiosis and fertility in *Drosophila melanogaster*

**DOI:** 10.1038/s41598-024-53346-z

**Published:** 2024-02-13

**Authors:** Siqi Sun, Tyler Defosse, Ayla Boyd, Joel Sop, Faith Verderose, Diya Surray, Mark Aziz, Margaret Howland, Siwen Wu, Neha Changela, Janet Jang, Karen Schindler, Jinchuan Xing, Kim S. McKim

**Affiliations:** 1 Department of Genetics, Rutgers, The State University of New Jersey, Piscataway, NJ USA; 2 Waksman Institute, Rutgers, The State University of New Jersey, 190 Frelinghuysen Road, Piscataway, NJ 08854 USA; 3 Human Genetics Institute of New Jersey, Rutgers, The State University of New Jersey, 145 Bevier Road, Piscataway, NJ 08854 USA

**Keywords:** Functional genomics, Genomics, Experimental organisms, Germline development

## Abstract

Reproductive success requires the development of viable oocytes and the accurate segregation of chromosomes during meiosis. Failure to segregate chromosomes properly can lead to infertility, miscarriages, or developmental disorders. A variety of factors contribute to accurate chromosome segregation and oocyte development, such as spindle assembly and sister chromatid cohesion. However, many proteins required for meiosis remain unknown. In this study, we aimed to develop a screening pipeline for identifying novel meiotic and fertility genes using the genome of *Drosophila melanogaster*. To accomplish this goal, genes upregulated within meiotically active tissues were identified. More than 240 genes with no known function were silenced using RNA interference (RNAi) and the effects on meiosis and fertility were assessed. We identified 94 genes that when silenced caused infertility and/or high levels of chromosomal nondisjunction. The vast majority of these genes have human and mouse homologs that are also poorly studied. Through this screening process, we identified novel genes that are crucial for meiosis and oocyte development but have not been extensively studied in human or model organisms. Understanding the function of these genes will be an important step towards the understanding of their biological significance during reproduction.

## Introduction

Fertility requires both a successful meiosis to provide balanced genetic complement to offspring, and several developmental processes to make viable zygotes. During meiosis, germ line cells undergo a single round of genome duplication followed by two consecutive chromosomal divisions prior to fertilization. The meiotic process is highly regulated by multiple cellular structures and protein complexes, but the processes are error-prone, especially in oogenesis. In humans, chromosome segregation errors during oocyte meiosis increase with age. This increase could be related to the unique features of oocytes, such as the extended meiotic arrest, the absence of centrosomes, and possibly other endogenous and exogenous factors^1^. Failure to produce high-quality gametes leads to infertility, spontaneous abortions, and birth defects^2^. Many genes have been identified that are responsible for accurate meiotic divisions and regulate discrete cell-cycle phases, meiotic events, and gamete viability^3–6^. Nevertheless, we still lack a complete picture of the proteins that control key processes in meiosis, such as chromosome cohesion, chromosome biorientation, and spindle assembly.

In addition to meiosis, defects in numerous developmental processes can also lead to infertility, such as genes required for germline development^7^. Furthermore, in most animals, maternally derived gene products regulate the early events of embryogenesis^8^. These include genes required for egg activation, early mitotic divisions, and the maternal-to-zygotic transition^9–11^. Elimination of these genes in the mother can lead to loss of fertility. It is likely that many additional factors are responsible for the loss of female fertility in humans, although few have been identified^5,12^.

*Drosophila* is a simple but vital genetic model for identifying and understanding the function of genes required for germline and embryonic development and meiosis^13,14^. Despite the differences between *Drosophila* and human physiology, the homologs of many human genetic disease loci show selective expression in *Drosophila* tissues analogous to the affected human tissues^15^. Additionally, the ability to produce a large number of offspring in a short period of time makes *Drosophila* a powerful system for gene discovery and studying potential disease-causing genes^16,17^.

In *Drosophila*, many well-studied genes required for meiosis are upregulated within the ovary, such as components of the synaptonemal complex *c(2)M* and *c(3)G*^18,19^ and members of the Chromosome Passenger Complex such as INCENP, and Aurora B kinase^20^. Genes that are required for oocyte and early embryonic development are also upregulated in the ovary, such as *nanos*, *vasa*, and *bicoid*^21–24^. By considering a gene’s expression pattern and sequence homology, it is possible to predict the function and subcellular localization of novel proteins. Such information would allow prioritization of screens based on the likelihood of a gene being involved in a biological process such as meiosis. Several online databases provide tissue-specific expression profiles and functional annotations of *Drosophila* genome, and they could be used to identify potential genes required for meiosis and fertility^15,25–27^. In this study, we identified 94 novel genes involved in meiosis and fertility by combining tissue-expression profile, gene annotation, functional analysis with RNAi knockdown, and cytological analysis. When these genes were knocked down in the germline, the flies displayed meiosis or germline development related phenotypes, including sterility, reduced fertility, and elevated levels of nondisjunction. These genes are excellent candidates for future mechanistic studies in flies and mammals.

## Methods

### Candidate gene selection from FlyAtlas 1

Gene expression profiles in different fly tissues were downloaded from FlyAtlas 1 (http://flyatlas.org/data.html)^15^. A total of 18,770 probe sets from 13,500 genes were included on the Affymetrix *Drosophila* Genome 2 expression array. The gene names and Flybase IDs were validated and corrected by FlyBase (https://flybase.org/). Genes with the following symbols in their names were removed, including unknown genes (“---”), non-protein-coding genes (“CR”), RNA genes (“rna”), and ribosomal proteins (“rps”/“rpl”). Expressions in tissue “*Drosophila* S2 cells” were excluded from analysis.

For each gene, the enrichment value for each tissue was calculated as the tissue-specific mean expression divided by the mean of the fly whole body; the p value was calculated using two-tailed Student’s t test. The expression direction is categorized as “up” when the enrichment value is larger than 1 and the p value is less than or equal to 0.05 (enrichment > 1 and *p*-value ≤ 0.05), “down” as enrichment < 1 and *p*-value ≤ 0.05, and “others” for anything else. To select genes with high confidence, microarray probe sets with no signals detected among four biological replicates (i.e., no “present” calls) in ovary, testis, and larvae central nervous system (CNS) were removed. One representative probe set was selected for each gene by selecting the one with the most biological replicate results and direction being “up” in ovary, testis, and larvae CNS. The selected genes were divided in four groups: ovary_only (“up” in ovary expression and not “up” in other tissues), ovary + cns (“up” in ovary and larvae CNS expression, not “up” in other tissues), ovary + testis (“up” in ovary and testis expression, not “up” in other tissues), and ovary + CNS + testis (“up” in ovary, larvae CNS, and testis expression, not “up” in other tissues).

### Candidate gene annotation and prioritization for experimental validation

To identify novel meiosis gene for functional validation, gene symbols with the format “CG + numbers” were considered to be not-well studied and selected. Next, the transgeneic RNA interference line for the candidate genes were searched in the Transgenic RNAi Project (TRiP)^28,29^ at Bloomington Drosophila Stock Center (https://bdsc.indiana.edu/index.html). Candidate genes without TRiP stocks were removed.

Experimentally-validated and predicted meiosis genes in *Drosophila* were extracted from MeiosisOnline (https://mcg.ustc.edu.cn/bsc/meiosis/index.html)^30^. Gene expression value of three ovary cell clusters, germ cells (GC), germarium soma, and follicle cells (FC), were calculated using single-cell transcriptome data^31^. For each cluster, the expression level of each gene was calculated as the median value of expressions from individual stages as defined in the Supplemental Table S2 of^31^.

### Candidate gene annotation, enrichment and protein–protein interaction network analyses

Orthologs and known alleles of candidate genes were annotated using FlyBase^27^. Enrichment analyses were performed for positive genes with human and mouse orthologs using the overrepresentation analysis in ConsensusPathDB (CPDB, http://cpdb.molgen.mpg.de/)^32^. Enriched terms (e.g., Gene Ontology, pathway, or protein complex) containing at least two input genes were selected for further analysis. Enrichment p values were determined by CPDB using a hypergeometric test and q values represent the adjusted p values using the false discovery rate method.

Protein–protein interaction (PPI) network of positive genes was constructed using interactions information provided by STRING *Drosophila melanogaster* database^33^. Interactions with at least medium confidence (i.e., combined scores ≥ 0.4) were selected.

### RNAi knockdown, sterility and nondisjunction (NDJ) assays, and RT-PCR

Potential meiotic genes were screened using RNA interference (RNAi). Stocks for RNAi were obtained from the Bloomington Drosophila Stock Center at the Indiana University Bloomington (https://bdsc.indiana.edu/index.html). In these transgenes, the shRNA sequence is placed downstream of the UAS enhancer, thus requiring the presence of the GAL4 activator to begin gene knockdown via RNAi.

**Figure 1:**
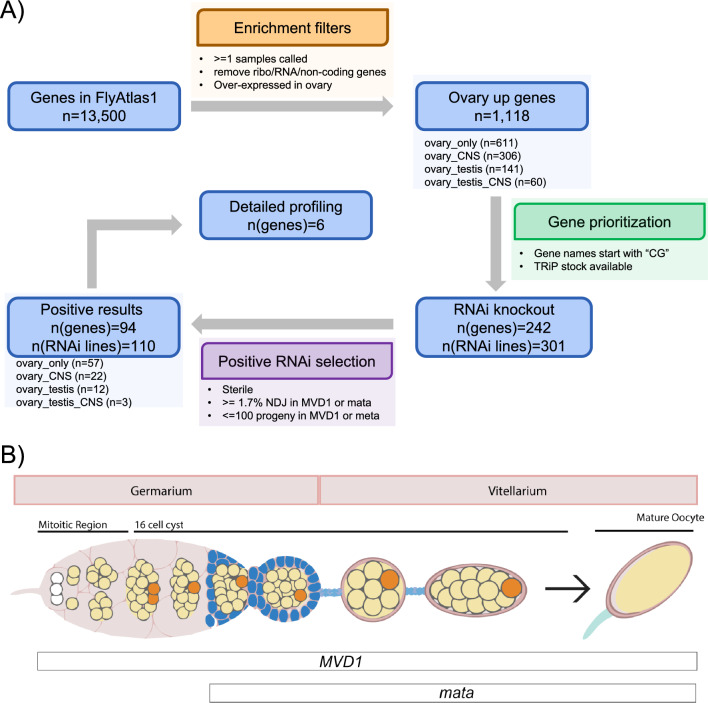
Analysis workflow. (**A**) The original 13,500 genes in Flyatlas1 were first selected by different enrichment and prioritization filters. The passed genes were validated by RNAi knockdown and sterility and nondisjunction assays. A total of 94 genes and the associated 110 RNAi lines were identified as positive meiosis genes. The numbers of genes in different sub-categories (e.g., ovary_only) were counted. The numbers of genes (n(genes)) and RNAi lines (n(RNAi lines)) after each filter were denoted. (**B**) Schematic of the *Drosophila* ovary and expression patterns of *P{GAL4: :VP16-nos.UTR}CG6325*^*MVD1*^ (referred to as *MVD1*) and *P{w[*+ *mC]* = *matalpha4-GAL-VP16}V37* (referred to as *matα*).

Crosses were set up using 10–20 *UAS:shRNA* males and 15–25 females with a tissue-specific *GAL4*. Each shRNA was crossed to two *GAL4* stocks to induce expression (Fig. 1B). *P{GAL4: :VP16-nos.UTR}CG6325*^*MVD1*^ (referred to as *MVD1*) initiates expression of transgenes in the pre-meiotic mitotic cells of the germline and continues throughout oocyte development^34^. *P{w[*+ *mC]* = *matalpha4-GAL-VP16}V37* (referred to as *matα*) expression begins late in prophase (region 2b/3), after early pachytene and the formation of crossovers and the synaptonemal complex, and continues through late prophase until stage 14 oocyte^20,35^.

All crosses were kept at 25 °C and were allowed to incubate for 10 days until progeny began to emerge. Virgin female progeny from this cross that carried both the UAS RNAi and the *GAL4*, as indicated by the y + and w + phenotypes, were collected. Five *UAS RNAi/GAL4* females were then crossed to five *y w/Y, B*^*s*^ males in five sets of vials (replicates). On days 14 and 18, the crosses were scored by recording the number of wildtype females (X/X), Bar males (X/B^S^ Y), aneuploid males (X/O), and aneuploid females (X/X/B^S^ Y). The frequency of NDJ was calculated as 2(XO + XXY)/[2(XO + XXY) + X/X + X/Y]. An RNAi line is considered positive if the cross has at least one of the following phenotypes: 1) sterile; 2) NDJ frequency ≥ 1.7% in either *MVD1* or *matα*, or 3) ≤ 100 progeny in either *MVD1* or *matα*. A candidate gene is considered a positive gene if at least one of its shRNA lines is positive. Some positives from these two tests were crossed to *P{w[*+ *mC]* = *tubP-GAL4}LL7* (referred to as *Tub-Gal4*), which expresses throughout the whole body of the fly and is a test for a somatic function such as mitosis. For several candidate genes, mutations were available from either the Bloomington Drosophila Stock Center or the National Institute of Genetics Fly Stocks (the NIG-Fly sgRNA and KO collections).

For reverse transcriptase quantitative PCR (RT-qPCR), total RNA was extracted from late-stage oocytes using TRIzol® Reagent (Life Technologies). cDNA was consequently prepared using the High Capacity cDNA Reverse Transcription Kit (Applied Biosystems). The qPCR was performed in a StepOnePlus™ (Life Technologies) PCR system using TaqMan® Gene Expression Assays (Life Technologies).

### Cytology

To study the effects of target gene knockdowns on germline development, meiosis, and embryonic development, the gross morphology of the ovaries was examined using a dissecting microscope. Small ovaries are indicative of a failure in germline development, such as loss of germline stem cells or development of the ovarian cysts. For cytological examination of early (germarium/ pachytene) or late (stage 14/Metaphase I) meiosis, oocytes were collected and examined using immunofluorescence. Crosses were set up using 10–20 *UAS:shRNA* males and 15–25 tissue-specific *GAL4* females. Twelve days following the cross, approximately 20 (for germarium) or ~ 300 (for stage 14) progeny that carried both the UAS regulated RNAi (y+) and the tissue-specific *GAL4* (w+) were collected and fed yeast for 2 days at 25 °C to promote egg laying. After 2 days, oocytes were collected and fixed using whole mounts to preserve the three-dimensional structure. Details of the two fixation protocols are described elsewhere^36,37^.

The primary antibodies used were: mouse anti-C(3)G (1:500)^38^, rabbit anti-C(2)M (1:400)^18^, a combination of two mouse anti-ORB antibodies (4H8 and 6H4, 1:100)^39^, rat anti-VASA (1:200), rabbit anti _γ_H2AV (1:500)^40^, mouse anti-α-tubulin conjugated to FITC (1:50) to stain microtubules, rabbit anti-CID (1:1000), and rat anti-INCENP (1:600). Additionally, Hoechst 33342 was used to stain for DNA. Following overnight incubation, oocytes were washed and stained with secondary antibodies for 4 h at room temperature. The secondary antibodies used were: goat anti-rat Cy3 (1:100), goat anti-rabbit 647 (1:100), and Alexa 488 (1:200) (Jackson Labs and Invitrogen). Oocytes were then mounted in SlowFade Gold (Invitrogen) and imaged using a Leica TCS SP8 confocal microscope with a 63x, NA 1.4 lens. All images shown are maximum projections of complete image stacks. Statistical analysis of sister kinetochore separation and foci quantification were performed using the microscopy image analysis software Imaris (Oxford Instruments).

## Results

### Selection of genes up-regulated in the ovary

FlyAtlas 1 contains mRNA expression levels of 13,500 genes in *Drosophila* adult and larval tissues. We selected 10,948 genes that are expressed in ovary, testis, and larval CNS. Genes that were up-regulated in adult ovaries while not up-regulated in other tissues except testis and larval CNS were selected as initial genes of interest (see Methods for detail). This selection resulted in 1118 genes, 611 of which were up-regulated only in ovary (referred to as “ovary-specific” in the following text). The ovary-specific genes included known meiotic genes, such as *c(2)M* and *c(3)G.* Among the 1118 genes, 141 were up-regulated in both ovary and testis. These genes were included because testis is a meiotic tissue. Genes in this selection included *ord,* a gene required for sister chromatid cohesion. Finally, because of mechanistic conservation in segregating chromosomes, we included genes required for meiosis that are also required for mitosis. Therefore, we included genes up-regulated in ovary and larval CNS because the larval CNS is a mitotically active tissue. Among genes upregulated in ovary, 306 were also up-regulated in CNS and 60 were up-regulated in both CNS and testis (Fig. 1A). Examples of these genes included spindle-associated proteins such as the CPC components *Incenp* and aurora kinase B (*aurB*), and kinetochore proteins such as *Ndc80* and *Spc105R.*

Among the 1118 genes, 974 have human orthologs and 964 have mouse orthologs (Table S1). Importantly, 873 of these genes have a shRNA TRiP stock which makes phenotype screening feasible^28^. To identify potential novel meiosis genes, we then selected a subset of uncharacterized genes to examine, many of which lack a gene name, and for whom a shRNA TRiP stock was available. This group was enriched for genes that have not been studied before and have no known function. After further manual review, we selected 242 genes for fertility and non-disjunction assessment after RNAi knockdown (Fig. 1A). Since beginning this study some genes that were only known by a CG name have since been named (Table 1, Table S2).

**Table 1:** 94 meiosis-related genes RNAi knockdown phenotypes. See Table S2 for a full list of tested RNAi lines and genes.

Gene number	Gene symbol	Human ortholog	Mouse ortholog	TRiP#	MVD1	Matα	No stage14	Tub-Gal4
*CG10050*	*Dtwd2*	*DTWD2*	*Dtwd2*	GL01481	505, 0 NDJ	119, 1 NDJ		
*CG10336*	*CG10336*	*TIPIN*	*Tipin*	HMJ22595	610, 0 NDJ	55, 0 NDJ		
*CG10336*	*CG10336*	*TIPIN*	*Tipin*	GLC01611	STERILE	6, 0 NDJ		Viable
*CG10344*	*CG10344*	*ZDHHC2*	*Zdhhc25*	HMS00197	318, 0 NDJ	119, 8 NDJ		
*CG10635*	*Pfdn4*	*PFDN4*	*Pfdn4*	HMC06544	526, 1 NDJ	42, 2 NDJ		Viable
*CG10981*	*dgrn*	*RNF4*	*Rnf4*	GL00588	565, 5 NDJ	903, 3 NDJ		
*CG11133*	*BoYb*	*DDX46*	*Ddx46*	HMJ23886	STERILE	STERILE		Viable
*CG11188*	*Aatf*	*AATF*	*Aatf*	HMC04594	STERILE	STERILE		
*CG11660*	*RIOK1*	*RIOK1*	*Riok1*	GL00195	175, 0 NDJ	STERILE	Matα	Lethal
*CG11660*	*RIOK1*	*RIOK1*	*Riok1*	HMC04524	STERILE	STERILE		
*CG11674*	*CG11674*	*DKK4*	*Dkk1*	HMS04336	819, 2 NDJ	50, 0 NDJ		
*CG11985*	*Sf3b5*	*SF3B5*	*Sf3b5*	HMS00097	STERILE	32, 0 NDJ		
*CG12018*	*PolD2*	*POLD2*	*Pold2*	HMC06042	694, 0 NDJ	95, 1 NDJ		Viable
*CG12077*	*PIG-C*	*PIGC*	*Pigc*	HMC06127	354, 0 NDJ	99, 0 NDJ		
*CG12179*	*Alms1a*	*notfound*	*notfound*	HMJ30289	ND	16, 0 NDJ		
*CG12259*	*CG12259*	*FAM50A*	*Fam50a*	HMJ23711	534, 22 NDJ	386, 2 NDJ		Viable
*CG1239*	*CG1239*	*MEPCE*	*Mepce*	HMS02605	STERILE	52, 0 NDJ		Lethal
*CG1239*	*CG1239*	*MEPCE*	*Mepce*	HMS02507	212, 0 NDJ	30, 0 NDJ		
*CG1239*	*CG1239*	*MEPCE*	*Mepce*	HMS02738	STERILE	STERILE		
*CG1239*	*CG1239*	*MEPCE*	*Mepce*	HMC02896	STERILE	96, 0 NDJ		Lethal
*CG13096*	*CG13096*	*RSL1D1*	*Rsl1d1*	HMS00206	STERILE	41, 0 NDJ		
*CG13690*	*CG13690*	*RNASEH2A*	*Rnaseh2a*	HMC03555	43, 0 NDJ	STERILE		Viable
*CG13741*	*Boot*	*notfound*	*notfound*	GL00570	STERILE	76, 1 NDJ		Lethal
*CG13773*	*Polr1F*	*TWISTNB*	*Twistnb*	HMC05572	STERILE	167, 0 NDJ		
*CG14174*	*CG14174*	*NEPRO*	*Nepro*	HMJ30042	STERILE	STERILE	MVD1, Matα	Lethal
*CG14230*	*CG14230*	*NOL8*	*Nol8*	GLC01782	257, 1 NDJ	STERILE	Matα	Lethal
*CG14303*	*qin*	*TDRD5*	*Tdrd5*	GL01398	52, 0 NDJ	468, 0 NDJ		Viable
*CG14931*	*CG14931*	*notfound*	*notfound*	GL00727	353, 0 NDJ	454, 4 NDJ		
*CG14962*	*Asciz*	*ATMIN*	*Atmin*	HMJ21116	292, 3 NDJ	397, 0 NDJ		
*CG15220*	*RPA3*	*RPA3*	*Rpa3*	HMJ24068	STERILE	STERILE	MVD1	Lethal
*CG15436*	*Paris*	*ZNF763*	*Zscan26*	HMC04637	485, 5 NDJ	175, 0 NDJ		
*CG15863*	*Pdrg1*	*PDRG1*	*Pdrg1*	HMC05965	STERILE	131, 0 NDJ		Viable
*CG1677*	*CG1677*	*ZC3H18*	*Zc3h18*	HMC04042	STERILE	STERILE		Viable
*CG16838*	*elg1*	*CRLF3*	*Crlf3*	HMC03187	STERILE	STERILE	Matα	Leaky
*CG16838*	*elg1*	*CRLF3*	*Crlf3*	HMJ30117	59, 0 NDJ	STERILE	Matα	Leaky
*CG16892*	*Aladin*	*AAAS*	*Aaas*	HMC03342	541, 1 NDJ	39, 0 NDJ		
*CG17233*	*CG17233*	*CCDC82*	*Ccdc82*	HMJ22836	462, 0 NDJ	24, 0 NDJ		
*CG17361*	*CG17361*	*notfound*	*notfound*	HMC04903	418, 0 NDJ	STERILE		Viable
*CG17658*	*CG17658*	*CHAT*	*notfound*	GL01020	396, 1 NDJ	284, 3 NDJ		
*CG17829*	*Hinfp*	*HINFP*	*Hinfp*	GL01162	352, 8 NDJ	229, 4 NDJ		Viable
*CG18586 /// CG5568*	*CG5568*	*ACSBG2*	*Acsbg2*	HMJ23127	ND	99, 0 NDJ		
*CG18787 /// CG18789*	*CG18787*	*NUP42*	*Nupl2*	HMC03818	STERILE	19, 0 NDJ	MVD1	Viable
*CG18787 /// CG18789*	*CG18787*	*NUP42*	*Nupl2*	HMC04063	466, 1 NDJ	55, 0 NDJ		Viable
*CG2051*	*Hat1*	*HAT1*	*Hat1*	HMS01210	STERILE	STERILE	MVD1	
*CG2199*	*CG2199*	*ZFP92*	*Zfp92*	HMJ30228	STERILE	136, 0 NDJ		
*CG2924*	*CG2924*	*UBE2Q1*	*Ube2dnl2*	GLC01774	428, 1 NDJ	26, 1 NDJ		
*CG30020*	*CG30020*	*ZFP92*	*Zfp92*	HMJ22046	STERILE	289, 0 NDJ	MVD1	Viable
*CG30467*	*CG30467*	*SAAL1*	*Saa4*	HMC04317	ND	227, 2 NDJ		
*CG31998*	*CG31998*	*PRRT3*	*Prrt3*	GL01256	155, 0 NDJ	27, 0 NDJ		
*CG32344*	*CG32344*	*DDX54*	*Ddx54*	HMJ23354	STERILE	STERILE		Lethal
*CG33217*	*CG33217*	*PELP1*	*Pelp1*	GL01509	STERILE	STERILE	Matα	Lethal, F1
*CG33217*	*CG33217*	*PELP1*	*Pelp1*	HMS02699	STERILE	STERILE		Lethal, F1
*CG33228*	*CG33228*	*RBM28*	*Rbm28*	HMC04060	539, 0 NDJ	119, 1 NDJ		
*CG3407*	*CG3407*	*ZNF692*	*E4f1*	HMC04955	409, 1 NDJ	400, 15 NDJ		
*CG34261*	*CG34261*	*OARD1*	*Oard1*	HMC06638	386, 12 NDJ	177, 0 NDJ		
*CG3430*	*CG3430*	*MCMBP*	*Mcmbp*	HMC06551	586, 2 NDJ	226, 18 NDJ	Matα	Lethal
*CG3430*	*CG3430*	*MCMBP*	*Mcmbp*	GL01184	42, 0 NDJ	STERILE	Matα	Lethal
*CG3680*	*HIPP1*	*CDYL2*	*Cdyl2*	HMC05150	536, 0 NDJ	159, 5 NDJ		
*CG40042*	*Tim23*	*TIMM23*	*Timm23*	HMC06553	STERILE	STERILE		Lethal
*CG42232*	*CG42232*	*MAP1A*	*Mki67*	GL01022	STERILE	720, 3 NDJ		
*CG42307 /// mus312*	*mus312*	*SLX4*	*Slx4*	HMS00381	STERILE	272, 0 NDJ		Lethal
*CG42388*	*Nost*	*NOSTRIN*	*Nostrin*	GL01145	ND	STERILE		
*CG4554*	*CG4554*	*UTP20*	*Utp20*	HMC03176	196, 0 NDJ	STERILE		Lethal
*CG4730*	*CG4730*	*ZNF362*	*Zfp362*	HMC05139	524, 0 NDJ	260, 4 NDJ		
*CG4771*	*vret*	*TDRD1*	*Snd1*	GL00653	459, 1 NDJ	59, 0 NDJ		
*CG4849*	*CG4849*	*EFTUD2*	*Eftud2*	HMS01994	592, 2 NDJ	90, 0 NDJ		
*CG4857*	*tyf*	*notfound*	*notfound*	HMS05700	629, 6 NDJ	718, 2 NDJ		
*CG4936*	*CG4936*	*ZSCAN4*	*Zscan4c*	HMC05569	STERILE	39, 0 NDJ		
*CG4951*	*CG4951*	*notfound*	*notfound*	GL01154	STERILE	STERILE		Viable
*CG4973///DpseGA18564*	*mdlc*	*RNF113A*	*Rnf113a2*	HMS04472	STERILE	86, 0 NDJ		
*CG4980*	*BCAS2*	*BCAS2*	*Bcas2*	HMJ22596	271, 3 NDJ	150, 0 NDJ		
*CG5131*	*CG5131*	*ATP23*	*Atp23*	HMC06573	449, 0 NDJ	31, 0 NDJ		
*CG5235*	*CG5235*	*MOXD1*	*Moxd1*	GL01241	591, 6 NDJ	95, 1 NDJ		
*CG5626*	*Tsen54*	*TSEN54*	*Tsen54*	GL00247	247, 0 NDJ	54, 0 NDJ		
*CG5757*	*CG5757*	*CMPK2*	*Dtymk*	HMJ23271	STERILE	8, 0 NDJ		Lethal
*CG5877*	*CG5877*	*NRDE2*	*Nrde2*	HMC03904	STERILE	233, 2 NDJ	MVD1	Viable
*CG6227*	*Prp5*	*DDX46*	*Ddx46*	HMJ24050	STERILE	80, 0 NDJ		
*CG6540*	*Nup35*	*NUP35*	*Nup35*	HMC06300	304, 0 NDJ	STERILE	Matα	Lethal
*CG6610*	*CG6610*	*LSM5*	*Lsm5*	HMS04506	138, 0 NDJ	180, 3 NDJ		
*CG6693*	*CG6693*	*DNAJC9*	*Dnajc9*	GL01534	231, 0 NDJ	438, 5 NDJ		Viable
*CG6843*	*CG6843*	*CIR1*	*Cir1*	HMC05138	ND	257, 12 NDJ		Viable
*CG6937*	*CG6937*	*PABPN1L*	*Pabpn1l*	HMC04778	455, 17 NDJ	540, 2 NDJ		
*CG6937*	*CG6937*	*PABPN1L*	*Pabpn1l*	GL01252	STERILE	STERILE		Leaky
*CG6951*	*CG6951*	*DCTD*	*Dctd*	HMS03371	627, 1 NDJ	STERILE		Viable
*CG6951*	*CG6951*	*DCTD*	*Dctd*	HMS03380	154, 0 NDJ	50, 0 NDJ		Lethal
*CG7033*	*CCT2*	*CCT2*	*Cct2*	HMS01190	ND	STERILE		Lethal, F1
*CG7156*	*CG7156*	*RPS6KL1*	*Rps6kl1*	GL00155	643, 1 NDJ	367, 4 NDJ		
*CG7185*	*Cpsf6*	*CPSF7*	*Cpsf7*	HMS00113	STERILE	STERILE		Lethal
*CG7357*	*Odj*	*GFI1B*	*Zfp518b*	HMJ23468	STERILE	621, 0 NDJ		Lethal
*CG7357*	*Odj*	*GFI1B*	*Zfp518b*	HMC05191	STERILE	265, 0 NDJ		
*CG7372*	*Phs*	*ZNF20*	*BC025920*	HMC05211	434, 0 NDJ	77, 0 NDJ		
*CG7386*	*CG7386*	*ZNF668*	*Zfp668*	HMC05188	460, 0 NDJ	82, 0 NDJ		Viable
*CG7609*	*Wdr24*	*WDR24*	*Wdr24*	HMJ23816	631, 1 NDJ	58, 0 NDJ		
*CG7730*	*CG7730*	*notfound*	*notfound*	GL01109	53, 0 NDJ	37, 0 NDJ		
*CG7971*	*Srrm234*	*SRRM2*	*Srrm2*	HMC03678	STERILE	STERILE		Lethal
*CG7993*	*Non3*	*RPF2*	*Rpf2*	HMS02628	STERILE	98, 0 NDJ		Viable
*CG8116*	*TMEM216*	*TMEM80*	*Tmem80*	HMJ21848	705, 2 NDJ	41, 0 NDJ		
*CG8142*	*CG8142*	*RFC4*	*Rfc4*	HMJ02051	STERILE	116, 0 NDJ	MVD1	Viable
*CG8142*	*CG8142*	*RFC4*	*Rfc4*	GL00569	STERILE	STERILE		Lethal
*CG8173*	*CG8173*	*PBK*	*Pbk*	GL01801	156, 0 NDJ	21, 0 NDJ		
*CG8173*	*CG8173*	*PBK*	*Pbk*	HMS05007	425, 0 NDJ	326, 3 NDJ		
*CG8232///Rcp*	*Rbbp5*	*PAN2*	*Pan2*	GLC01808	421, 0 NDJ	101, 1 NDJ		
*CG8298*	*CG8298*	*GK3P*	*Gk2*	GL00301	514, 1 NDJ	20, 0 NDJ		
*CG8435*	*CG8435*	*YJU2*	*Yju2*	GL00696	STERILE	431, 0 NDJ		Viable
*CG8435*	*CG8435*	*YJU2*	*Yju2*	GL00696	STERILE	46, 0 NDJ		
*CG8728*	*CG8728*	*PMPCA*	*Pmpca*	HMS00561	STERILE	STERILE		
*CG8915*	*CG8915*	*YTHDC2*	*Ythdc2*	HMS00697	897, 10 NDJ	493, 1 NDJ		Viable
*CG8950*	*CG8950*	*GTF3C3*	*Gtf3c3*	HMC02421	526, 1 NDJ	271, 5 NDJ		
*CG8950*	*CG8950*	*GTF3C3*	*Gtf3c3*	HMJ22532	325, 0 NDJ	297, 5 NDJ		
*CG9203*	*mh*	*SPRTN*	*Sprtn*	GLC01597	ND	STERILE		Lethal

### Ovary-specific RNAi knockdown of candidate genes

Each *shRNA* strain was crossed to one of two GAL4 expressing females to induce shRNA expression (Fig. 1B). *P{GAL4: VP16-nos.UTR}CG6325*^*MVD1*^ (referred to as *MVD1*) initiates expression of transgenes specifically in the pre-meiotic cyst cells of the germline and expression continues throughout oocyte maturation^34^. *P{w[*+ *mC]* = *matalpha4-GAL-VP16}V37* (referred to as *matα*) expression begins in prophase (region2b/3) and expression continues through Metaphase I in stage 14 oocytes^20,35^. A significant phenotype (a “positive”) was recorded if the RNAi knockdown produced sterile females, or an increased nondisjunction (NDJ) rate (≥ 1.7%) that was higher than most of RNAi lines (Figure S1), or a small brood size (< 100). Because each gene was tested with *MVD1* and/or *matα* promoters, and because some genes were tested using multiple shRNA lines, we defined a positive gene as one with at least one RNAi experiment yielding a positive result with either the *MVD1* or the *matα* promoter.

**Figure 2:**
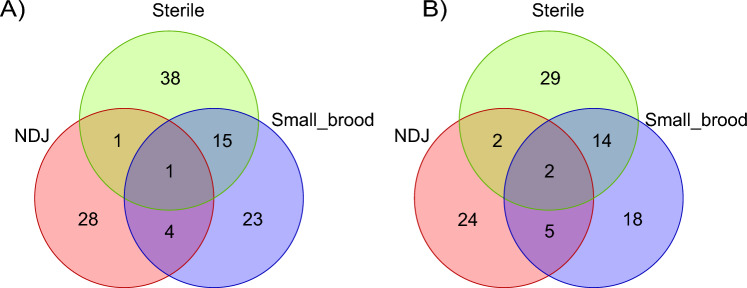
Venn diagrams for (**A**) 110 positive RNAi lines and (**B**) 94 positive genes. Some RNAi lines and associated positive genes are present in multiple phenotype categories (NDJ, Sterile, and small brood size (Small_brood)) because: (1) different phenotypes were observed in the MVD1 and matα experiments; (2) phenotypes of some lines met the criteria of both NDJ and small brood size. For example, RNAi line *GL00570* and the associated *CG13741* gene was Sterile in *MVD1* and meet both NDJ and small brood size criteria in *matα*. In addition, *CG3430* was tested with multiple RNAi lines and are present in all phenotype categories. See details in Table 1.

Following completion of nondisjunction and sterility assays on 242 novel gene candidates in 301 RNAi lines (Table S2), we identified 94 genes that showed evidence of a function in germline development, meiosis, or embryonic development in experiments with 110 RNAi lines (Table 1, Fig. 2). Some positive RNAi lines (e.g., GL00570) were present in multiple phenotype categories (NDJ, Sterile, and small brood size) because: (1) different phenotypes were observed between the *MVD1* and the *matα* experiments; (2) phenotypes of some shRNAs met the criteria of both NDJ and small brood size (Fig. 2A). Among the 94 genes, 57 are ovary-specific genes, 22 are expressed in both ovary and larval CNS, 12 are expressed in both ovary and testis, and 3 are expressed in all three tissues. Knockdown of 29 genes produced sterile females, 24 genes had an increased nondisjunction rate (≥ 1.7%), and 18 genes produced small brood sizes (< 100) (Fig. 2B). The remaining 23 genes showed different phenotypes in *MVD1* and *matα* crosses and/or with different shRNAs (overlapping sections of the Venn diagram, Fig. 2B). For example, CG10336 *MVD1/*shRNA knockdown females were sterile, but had a small brood size in *matα/*shRNA females. The difference between the two *GAL4* lines provides temporal information on when these genes function during oocyte development.

To confirm that the RNAi approach was effective, five positive genes that produced sterile females were tested by qRT-PCR. All showed significant knockdowns of the intended genes (Table 2, *CG4951, CG18787, CG3430, CG10336, CG8142*).

**Table 2:** RT-PCR results.

Gene	shRNA	% of wild-type mRNA (%)
*CG4951*	*GL01154*	6
*CG18787*	*HMC03818*	34
*CG3430*	*GL01184*	0
*CG10336*	*GLC01611*	0.4
*CG8142*	*GL00569*	5

### Annotation and enrichment analyses of positive genes

To identify genes with a role in meiosis, we examined proposed functions of each identified gene in other species (Table 1). Among the 94 genes, 86 genes have both human or mouse orthologs. Using human or mouse orthologs of the *Drosophila* genes, we performed gene ontology (GO) and pathway enrichment analyses (Tables S3, S4). Several GO terms showed significant enrichment in human and mouse orthologs, such as replication fork (q < 5e^−5^ in human and mouse, Table S3). Similarly, several pathways showed significant enrichment, such as DNA replication (q < 2.7e^−4^ in mouse), DNA repair (q = 0.016 in mouse, q = 0.0024 in human, Table S4). These GO terms and pathways might be expected as important for various steps of meiosis or the mitotic divisions of the early embryo. In addition, genes associated with RNA biology, such as spliceosome complex, RNA helicase activity, and ribosome biogenesis, also appeared frequently in the analysis. These findings are consistent with the known importance of regulating RNAs in oocyte and early embryonic development^41–43^.

**Figure 3:**
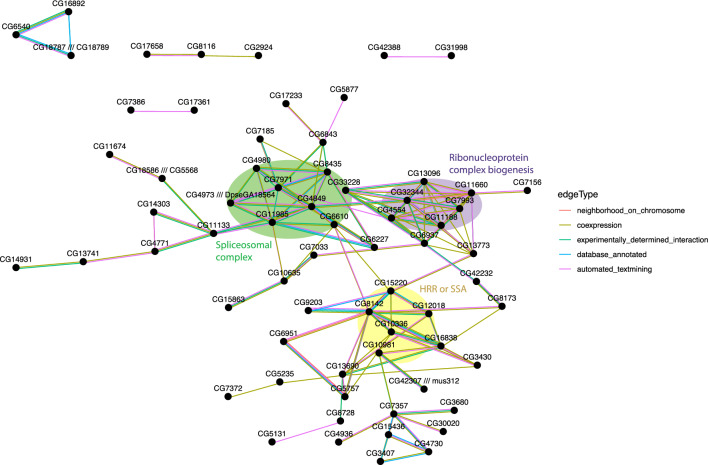
Protein–protein interaction network of candidate genes. Only connected genes are shown. Genes present in three representative enriched GO terms: ribonucleoprotein complex biogenesis, spliceosomal complex, and HDR through homologous recombination (HRR) or single-strand annealing (SSA) are highlighted with the enriched terms labeled. See Supplemental Table S5 for a full list of interactions.

Furthermore, we examined the interaction among the candidate genes by constructing a protein–protein interaction (PPI) network. We identified a network of 67 positive genes in one main cluster (Table S5, Fig. 3). The cluster contains genes in two significantly enriched GO terms related to cellular component biogenesis and mRNA splicing: ribonucleoprotein complex biogenesis (q < 3.1e^−5^ in mouse, *CG32344, CG4554, CG13096, CG11188, CG11660,* and *CG7993*) and spliceosome complex (q < 1.5e^−5^ in human, *CG8435, CG4980, CG33228, CG11985, CG6610, CG7971, CG4849, and CG4973*). These genes could be required for generating oocytes with enough maternal products to support embryonic development. We also found three genes in the pathway HDR through Homologous Recombination (HRR) or Single Strand Annealing (SSA) (*CG8142, CG10336, CG12018, CG15220,* and *CG10981*). These genes could be important for meiosis as homology-directed repair is crucial during meiosis to ensure proper chromosome segregation^6^.

While we selected genes expressed in the ovary, ovaries are a complex tissue consisting of somatic and germline cell types. Insights into the function of these genes could come from identifying in which ovarian cell type they are expressed. A single cell ovary transcriptome dataset has identified several somatic (germarium soma, follicle cell (FC)) and germline cell (GC) cell types in ovaries^31^. Based on this dataset, 907 of the 1118 ovary up-regulated genes in our initial dataset (81%) had higher expression in the GC cluster compared to the germarium soma and FC clusters, with 85% of positive genes (80 genes) showing the same trend. These results suggest that our candidate genes are highly enriched in germline-specific genes and are likely to function in oocyte development.

### Analysis of ovaries from select shRNA lines: gene with germline-specific phenotypes

Some genes are expected to be specific to the germline. These genes are predicted to have low expression in the central nervous system and be viable when the shRNA is expressed with whole-body promoter *Tub-Gal4.* Genes that fit this pattern are good candidates for germline-specific genes (Table 1)*.* Examples of germline-specific genes include *CG11133, CG18787, CG4951, CG5877* and *CG8435.* In some cases, the tissue gene expression data did not predict the somatic phenotype with *Tub-Gal4*. For example, *CG10336* encodes the orthologue of human *TIPIN*. Although expressed in the nervous system, shRNA *GLC01611* was viable when expressed with *Tub-Gal4.* This is the expected phenotype for genes that may not be required for ovary development unless there is an inducer of DNA damage^44^, which may include *CG10336*. In some other cases, *Tub-Gal4* crosses might not provide expected results. For example, if *Tub-Gal4* is provided maternally, then it could drive expression of the shRNA in embryo and cause embryonic lethality. Null alleles could survive, however, because the maternal contribution of wild-type protein allows them to survive. An example of a candidate in this class is *CG13741*, also known as *Bootlegger* (*Boot*). *Boot* is an ovary-specific gene, and null alleles are viable but female sterile^45^, although the shRNA expressed with *Tub-Gal4* was lethal. Similarly, null alleles of *CG9203* are viable but female sterile, but shRNA expressed with *Tub-Gal4* was lethal. *CG9203* is also known as *maternal haploid* (*mh*), a gene required for fusion of male and female pronuclei^46,47^. The roles of both of these genes in meiosis have not been studied.

### CG18787 knockdown has premature karyosome and sister centromere separation

One shRNA targeting *CG18787* could also target a second gene, *CG18789*. These two genes are located in close proximity to one another, separated by approximately 2.5 kb and two other genes. *CG18787* encodes a 398 amino-acid protein that has 99% amino acid identity with *CG18789*, with only 3 amino acid differences. Given the high similarity and the relative location of these two genes within the genome, it is likely that *CG18787* and *CG18789* arose from a recent gene duplication event. shRNA *HMC03818* produced a more severe fertility defect when crossed with both the *MVD1* and *matα* compared to shRNA *HMC04063* (Table 1). Analysis of the sequences of each shRNA demonstrated that *HMC03818* could target both *CG18787* and *CG18789*, while *HMC04063* probably targets only *CG18787*. Thus, *HMC03818* could cause a more severe fertility phenotype than *HMC04063* because its shRNA targets both genes. *HMC03818* did not cause lethality when crossed to *Tub-Gal4*, indicating *CG18787* may not be required for mitosis, and consistent with its low expression in the central nervous system.

**Figure 4:**
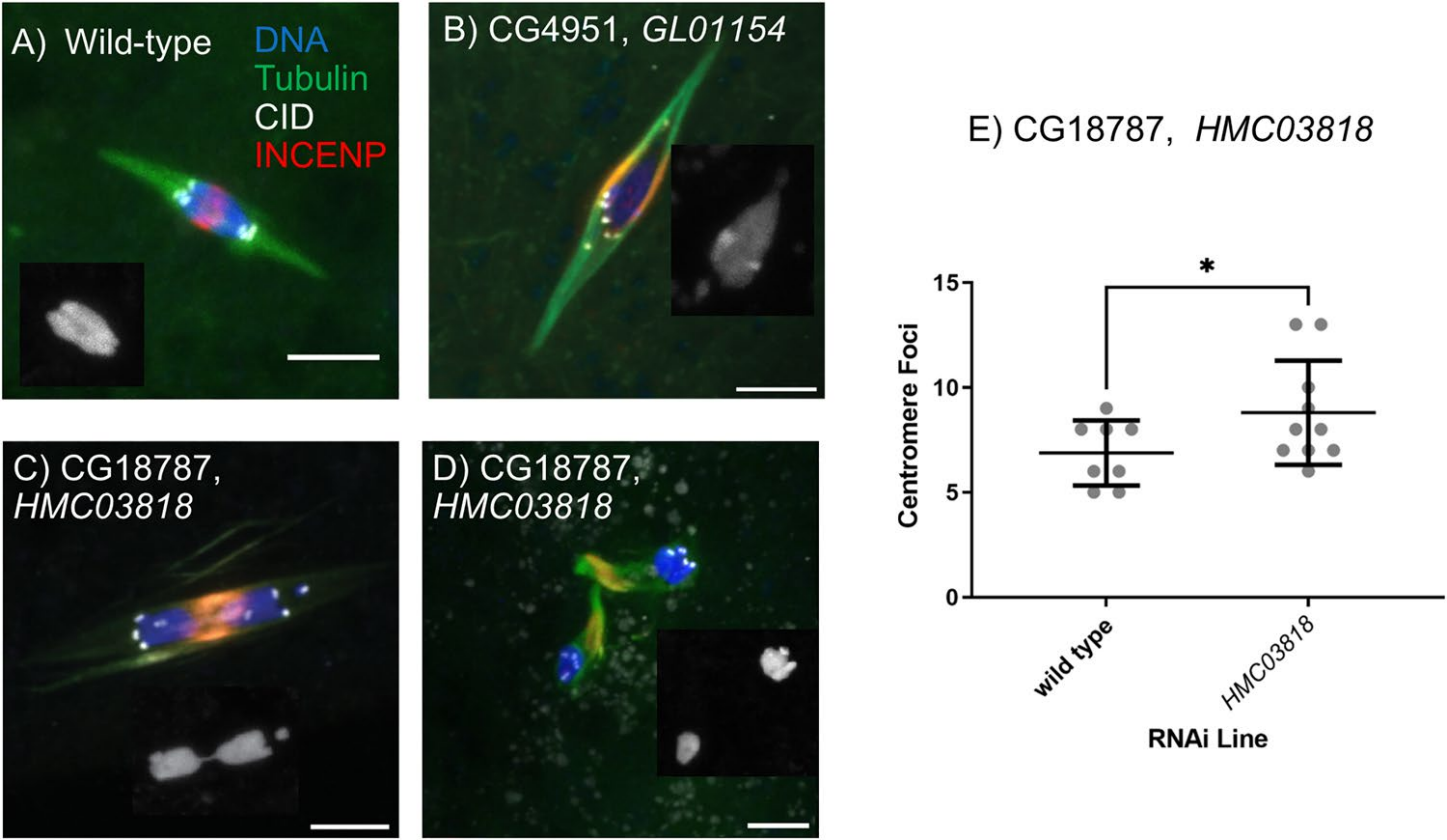
Cytological analysis of *Drosophila* oocytes. *Drosophila* oocytes were extracted and stained for Tubulin, the centromere (CID/CENP-A), central spindle (INCENP), and DNA (Hoechst). Scale bars are 5 μm. (**A**) An example of a wild-type Metaphase I arrested spindle with the formation of a singular karyosome and symmetric division of the centromeres. Knockdown using *matα* of (**B**) *CG4951* and (**C**,**D**) *CG18787*. Inset in each panel shows the karyosome (DNA). (**E**) Quantification of sister kinetochore foci for both wildtype and *CG18787* RNAi oocytes.

Mature oocytes expressing *HMC03818* had severe karyosome separation when compared to wildtype oocytes (54%, n = 24, wildtype: Fig. 4A; two examples of *HMC03818*: Fig. 4C,D). This severe phenotype is consistent with a defect in sister chromatid cohesion during meiosis^48,49^. In addition, the separation of sister kinetochores is a phenotype commonly associated with a loss of centromere cohesion^50^. Therefore, we determined the frequency of sister kinetochore separation in *HMC03818/ matα* oocytes. A normal meiosis is expected to have 8 foci, one for each pair of sister centromeres, although usually less is observed due to clustering of the centromeres. (avg = 6.9, Fig. 4E). In contrast, *HMC03818* RNAi oocytes had a significant elevated frequency of sister kinetochore separation (avg = 8.8, *p* = 0.0185, Fig. 4E). These results support the conclusion that *G18787* is required for sister chromatid cohesion.

**Figure 5:**
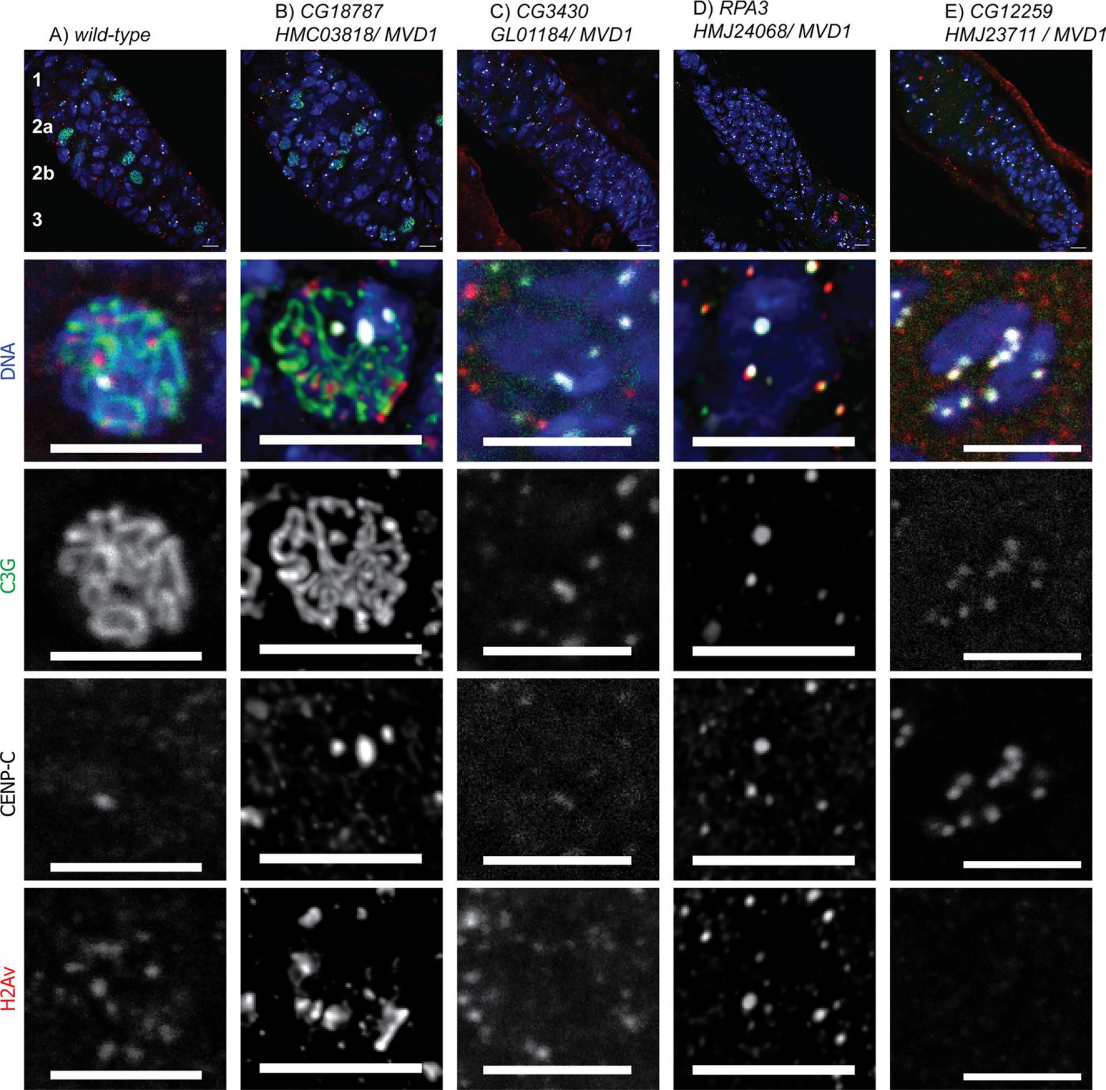
Early prophase (germarium) images. Ovaries were dissected from shRNA / *MVD1* females and stained for C(3)G (SC, green), _γ_H2AV (DSBs, red), CENP-C (centromeres, white), and DNA (blue). Panels: wild-type (**A**), RNAi against *CG18187* (**B**), *CG3430* (**C**), *RPA3* (**D**), and *CG12259* (**E**). The germarium is divided into four stages: 1 (mitotic region with stems cells, and 2, 4, and 8 cell cysts), 2 (16 cell cysts in early meiosis, including zygotene and pachytene), and 2b and 3 (mid pachytene 16 cell cysts). The ovaries in C-E lack 16 cell cysts. Some of the cells are somatic follicle cells and lack meiotic markers like C(3)G. The scale bars are 5 µm.

The cohesion defects were observed using *HMC03818/ matα,* and because meiotic cohesion is probably established during S-phase before *matα* expression^48^, these results suggest *G18787* has a cohesion maintenance function. In addition, mutants defective in establishing sister chromatid cohesion also have defects in Synaptonemal Complex (SC) assembly^51,52^. We examined early meiotic prophase oocytes from *HMC03818/MVD1* females and found no defects in SC component C(3)G and double-strand break (DSB) marker _γ_H2AV (wildtype: Fig. 5A; *HMC03818 / MVD1*: Fig. 5B). These results suggest that the defects associated with the loss of *CG18787* and *CG18789* may affect the maintenance of cohesion in oocytes. Mature oocytes were absent in *HMC03818 / MVD1* ovaries (Table 1), suggesting that *CG18787* is also required for the mitotic divisions of germline development.

*CG18787* contains a nucleoporin domain and has limited homology to human *NUP42*. This is significant because recent studies have found that upon nuclear envelope breakdown leading up to cellular division, many nucleoporins localize to kinetochores and serve alternative functions to aid in cellular division. An example of this is the nucleoporin *Elys*, which was found to function as a Protein Phosphatase 1 scaffold during M-phase exit and thus aids in the disassembly of kinetochores^53^. Based on these results, we believe that *CG18787* has a meiotic function required for the maintenance of sister chromatid cohesion.

### CG4951 may be required for biorientation

Knockdown of *CG4951* using shRNA *GL01154* caused sterility (Table 1). *GL01154* did not cause lethality when crossed to *Tub-Gal4*, suggesting the function of *CG4951* is germline-specific and not required for mitosis. This phenotype is consistent with its expression pattern, which shows low expression in the nervous system. Sequence analysis of this gene does not provide any significant insight into the function as the predicted protein sequence does not contain identifiable protein domains or regions of high conservation. *CG4951* is an example of a poorly conserved gene. It encodes a 320 amino-acid protein that is conserved within the *Drosophila* genus but not found in other Diptera such as the mosquito. Cytological analysis of *CG4951* RNAi oocytes did not reveal significant defects in spindle assembly (Fig. 4B). Further studies are needed to determine if there are biorientation defects, as we observed asymmetric distribution of centromeres, which would result in the abnormal division of the chromosomes at anaphase I (Fig. 4B, 2/10 oocytes). This phenotype would suggest that *CG4951* is involved in the regulation of kinetochore biorientation.

### Genes with somatic phenotypes

Genes expressed at high levels in the nervous system could have somatic phenotypes*.* Lethality in the *Tub-Gal4* experiment, however, identifies any gene with a function in somatic tissue (Table 1). For example, *CG16838* is *enhanced level of genomic instability 1* (*elg1*). We have confirmed that most homozygous mutants (*elg*^*1*^*/elg*^*2*^) are lethal, with some rare but sick survivors, consistent with previous studies^54^. Consistent with this result, the shRNA expressed with *Tub-Gal4* caused lethality, although there were a few sick survivors. Interestingly, we identified another gene that probably interacts with *elg1, CG8142,* also known as *RFC4* in human. This gene is also expressed at high levels in the nervous system and shRNA *GL00569* is lethal with *Tub-Gal4.* One possibility, based on sequence homology, is that these genes are required for DNA replication in the germline. This would explain the lack of oocytes with shRNA for *CG8142* expressed with *MVD1.* However, *elg1* shRNA expressed with *matα* also failed to make oocytes, which cannot be a DNA replication defect because there is none in maturing oocytes. Further work is required to understand the defects, similarities, and differences, between these two genes.

Additional examples of genes required in the germline and somatic cells include *CG15220*, also known as *RPA3*, which failed to make oocytes when depleted with *MVD1*, was sterile with *matα*, and was lethal with *Tub-Gal4*. *CG7185* is known as Cleavage and polyadenylation specific factor 6 (*Cpsf6*) and is an essential gene^55^, consistent with its high expression in ovaries and the larval nervous system. *CG7033* (Chaperonin containing TCP1 subunit 2 (*CCT2*)) is also a gene in this category but had a unique phenotype with *Tub-Gal4.* The crosses were sterile, indicating the F1 embryos died. A similar phenotype was observed with *CG33217,* which is orthologous to human *PELP1* (proline, glutamate and leucine rich protein 1). Using shRNA *GL01509,* the F1 progeny from a cross to *Tub-Gal4* were lethal. These genes have an unusual dosage sensitivity as this must represent expression induced by maternal contribution of *Tub-Gal4.*

### *CG3430* knockdown has biorientation defects and precocious anaphase I onset

Two shRNAs were associated with *CG3430* (Table 1). The first shRNA, *GL01184*, caused reduced fertility and a loss of developing oocytes (see details below) when expressed with *MVD1*, and was sterile when expressed with *matα.* The second shRNA, *HMC06551*, had little effect when expressed with *MVD1* and elevated levels of nondisjunction (15.9%) when expressed with *matα*. The differences in these phenotypes indicate that *GL01184* resulted in a stronger knockdown of mRNA than *HMC06551.* Both shRNAs caused lethality when expressed by *Tub-Gal4*, indicating this gene could be required for mitosis as well. Furthermore, two CRISPR alleles were obtained from NIG-FLY, and were lethal as trans heterozygotes (*CG3430*^*SK5*^*/CG3430*^*SK7*^).

By sequence comparison, we found that *CG3430* is homologous to the Mini-Chromosome Maintenance Complex Binding Protein (MCMBP) superfamily. For example, CG3430 has 37% identity with human MCMBP, which was identified as a protein that associates with and promotes assembly of the MCM2-7 complex^56,57^. Other functions have also been suggested. In *Xenopus*, MCMBP promotes disassembly of MCM complexes from chromatin^58^. *Arabidopsis thaliana* MCMBP/ETG1 appears to be needed for sister chromatid cohesion^59^. Our results with *matα* demonstrate that *Drosophila* MCMBP has a function after premeiotic S-phase in oocytes.

### Genes required for early germline development

As noted above, shRNA targeting *CG8142* or *CG3430* expressed with *MVD1* resulted in ovaries lacking developing oocytes. To identify defects early in germline development when *CG3430* was depleted, ovaries from *GL01184/MVD1* females were dissected and stained with antibodies that mark SC component C(3)G and the DSB marker _γ_H2AV. We observed a defect early in germline development. The ovaries failed to make 16 cell cysts, and oocytes with full SC formation and _γ_H2AV foci were not observed (Fig. 5C). Thus, *CG3430* is required prior to meiosis, for the stem cell divisions or the mitotic divisions that generate the 16 cell cysts. Similarly, *CG15220,* encoding the *Drosophila* orthologue of *Replication protein 3A* (*RPA3*), is required for germline development, as shown by the absence of oocytes with full SC formation and the DSB marker _γ_H2AV in *HMJ24068*/*MVD1* ovaries (Fig. 5D).

**Figure 6:**
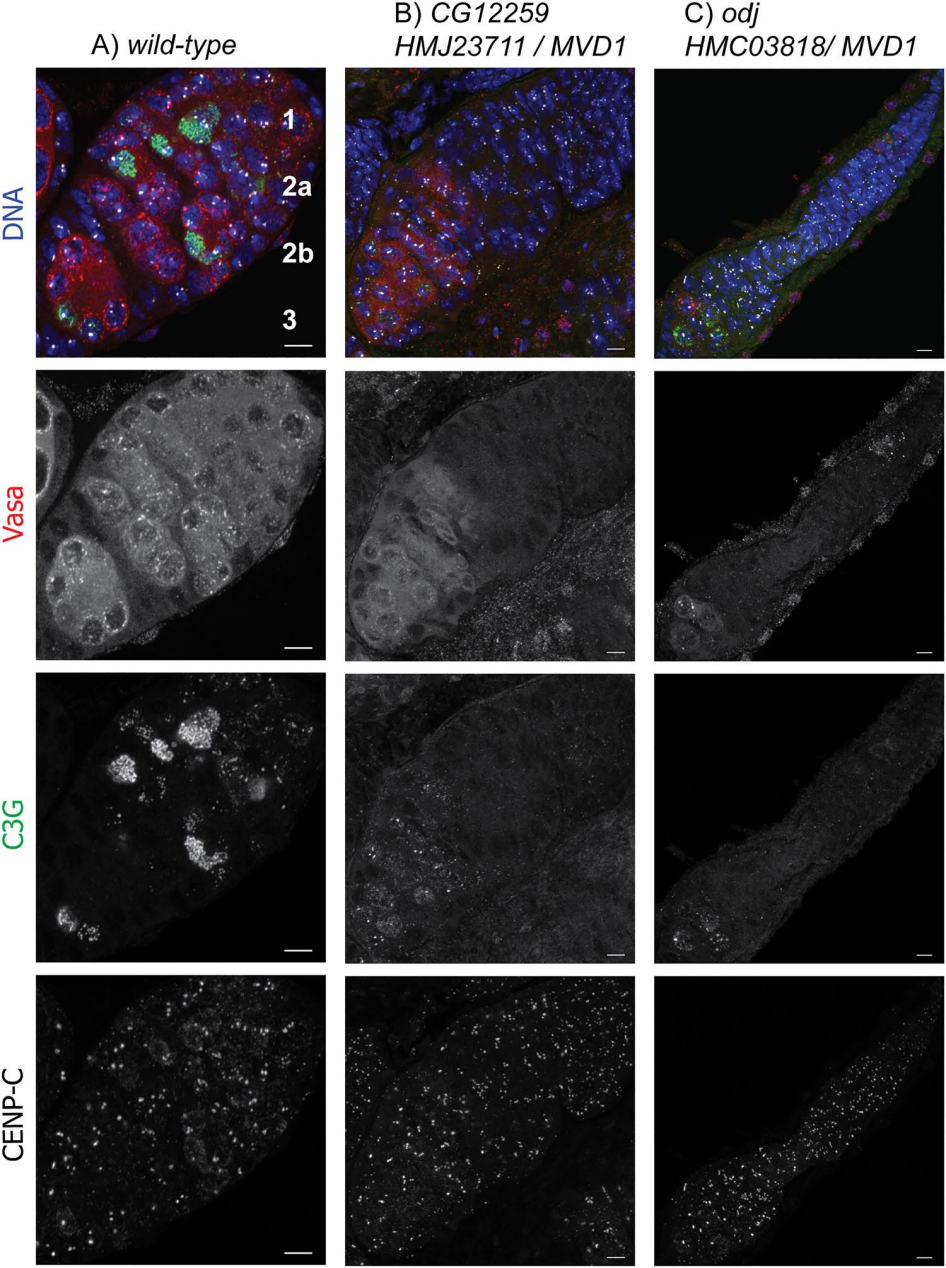
Whole germarium images. Ovaries were dissected from shRNA / *MVD1* females and stained for C(3)G (SC, green), VASA, (germline cells, red), CENP-C (centromere, white), and DNA (blue). Panels: wild-type (**A**), RNAi against (**B**) *CG1229* and (**C**) *Odj*. B and C have relatively small ovaries characterized by a lack of 16-cell cyst formation and C(3)G limited to the centromeres, indicating a loss of germ cells. The cells lacking VASA staining in A-C are somatic cells. The scale bars are 5 µm.

We observed a similar early germline defect with several other genes. Three lines of evidence suggest that *CG12259* is only required early in germ line development. First, *HMJ23711/MVD1* females were sterile, but *HMJ23711/matα* females were fertile. Second, cytological analysis of *HMJ23711/MVD1* females revealed a severe phenotype, with a defect early in germline development, and failing to make 16 cell cysts with an oocyte containing full SC formation or the DSB marker _γ_H2AV (Fig. 5E). We examined these ovaries with VASA staining, which is a marker specific for germline cells. Compared to wild-type ovaries, *HMJ23711/ MVD1* ovaries had a deficiency in cells with cytoplasmic VASA, indicating a loss of germline cells (wildtype: Fig. 6A; *HMJ23711/MVD1*: Fig. 6B). Thus, this gene may be required prior to meiosis, during the mitotic divisions that generate the 16 cell cysts. Indeed, the loss of germ cells in *HMJ23711/MVD1* ovaries is consistent with a defect in maintaining the stem cell population of the ovary. Third, *HMJ23711* did not cause lethality when crossed to *Tub-Gal4*, indicating *CG12259* may not be required for mitosis and is germline specific. *CG12259* has the highest homology with *FAM50A* in mammals with possible RNA/nucleic acid binding activity and functions in chromatin organization^60^.

Two shRNAs for *CG7357* (*HMC05191* and *HMJ23468*), also known as *Oddjob* (*Odj*)*,* had a similar phenotype in *MVD1* ovaries. *Oddjob* is a member of the ZAD family of zinc-finger transcription factors. There are no known mutations, although a similar result was observed using the same RNAi lines in a screen of ZAD transcription factors for functions in the female germline^61^. This function appears to be specific for the early germline because, like *CG12259, shRNA/matα* females were fertile. Also, like *CG12259, shRNA/ MVD1* ovaries lacked germ cells as shown by VASA staining, indicating *Oddjob* is required for maintaining the germline, possibly the stem cell population (Fig. 6C). However, *Oddjob* also has a somatic function, as suggested by its high expression in the nervous system and lethality of *HMJ23468* with *Tub-Gal4.*

## Conclusion

Sexual reproduction depends on two integrated processes, the faithful transmission of chromosomes during meiosis to yield viable gametes, and the development of a gamete capable of fertilization and supporting embryonic development. Our unbiased screen in *Drosophila* has identified candidate genes that could be further studied to expand our understanding of these critical processes.

In this study, we leveraged gene expression profiles and functional annotation to identify novel meiotic genes followed by phenotyping validation in *Drosophila* RNAi lines. We identified 94 genes that displayed elevated levels of nondisjunction or loss of fertility when knocked down, showing that these genes are required in multiple phases of gametogenesis and meiosis. We screened 301 RNAi lines using two *GAL4* promoters, which resulted in a selection for genes required early in gametogenesis or late in oogenesis. Only one of our 94 meiosis candidate genes (*CG42307///mus312*) were found in the manually curated meiosis database, MeiosisOnline^30^. This is probably because, by design, most of the genes we tested were not previously characterized. These results support our approach for identifying novel meiosis and fertility genes.

At least two previous studies used RNAi to screen for genes related to the loss of fertility. First, was a screen using a *GAL4* promoter with similar expression characteristics to *MVD1* and *matα* for maternal proteins that are phospho-regulated^62^. Among the 132 genes whose knockdown affected oogenesis (class 2 to class 6), three genes overlap our 1118 ovary up-regulated genes, including one of our positive genes *CG11188* and two genes that we tested but showed a normal phenotype (*CG6961, CG4968*). The most likely reason for lack of overlap is the different selection criteria, as focusing on a protein modification (phospho-regulated) may select for many genes that are not ovary enriched. Second was a screen using a *GAL4* similar to *MVD1* for genes required for germline stem cell maintenance^63^. Among the 366 positive genes, 10 overlaps with our positive results (*CG13096*, *CG8435*, *CG7033, CG8728, CG7185, CG8116, CG11985, CG11660, CG42307* and *CG30020*) and two overlaps with our negative results (*CG9548* and *CG11398*). The lack of overlap in this case is more surprising, but could be due to the screening phenotype (stem cell maintenance) compared to defects in fertility, or differences in how the genes for screening were selected.

The lack of overlap of these previous studies with our positive genes substantiates the validity of our approach of identifying novel genes. Future studies could continue novel gene discovery by integrating ovary up-regulated genes in FlyAtlas 1 and FlyAtlas 2^25^. Although there are differences in experimental design and data generation methods between the two databases, the majority of the FlyAtlas1 ovary up-regulated genes (72.7%) are also up-regulated in FlyAtlas2, including 66% of the positive genes. Another future direction would be to test these genes for a role in the male germline, using appropriate *Gal4* activators such as *MVD1* and *bam-GAL4.VP16*^64^.

The genes we discovered fall into a variety of functional classes. The use of the two *GAL4* lines to induce expression of the shRNA also provides useful temporal information. Some genes, such as *CG15863, CG5877, CG30020, CG42232,* and *CG7357*, were sterile with *MVD1* but not *matα*, indicating they had a function only in early germline development. Conversely, genes such as *CG17361, CG6951, CG6540*, and *CG4554* were sterile with *matα* but not *MVD1*, suggesting they only function late in oocyte development. A surprising number of genes produced no mature oocytes with *matα*. This phenotype with *matα*, such as *CG3430, CG14174, CG16838, CG33217,* and *CG6540*, suggests a role in growth of the oocyte during the meiotic prophase arrest. We also identified several genes with a reduced fertility phenotype. This could indicate a partial mRNA knockdown or a function important but not essential for fertility.

Several genes, such as *CG6937, CG3407, CG3430, CG12259, CG10635, CG10344, CG6843,* and *CG34261*, had significant nondisjunction phenotypes and thus appear to be required for meiosis. Although several genes involved in homolog pairing and recombination within *Drosophila* show little apparent sequence homology, there is strong genetic and structural conservation of meiosis across eukaryotes^65–68^. The functional similarity between human and fruit fly establishes the foundation that understanding of the *Drosophila* genome could provide a valuable source to yield insights into human gene functions that are not easily obtainable in mammals like humans or mice. For example, the positive gene *CG8915* is the homolog of a potential human meiotic gene *YTHDC2* (mouse Ythdc2), which plays a role in regulating meiosis in human and mouse^69–71^.

An important outcome of this screen is the identification of genes with no known ovary function that have homologs in higher eukaryotic systems. For example, 86 of the 94 genes have mouse homologs, and about one-third of these genes have no known function in meiosis or reproduction likely because they have not yet been examined. The high percentage of human/mouse homologs in our positive genes demonstrate this approach enriches for genes that are crucial for oocyte and embryo development and can uncover novel mechanisms for female infertility. This screen also uncovered nearly 20 genes that either function or have predicted function in RNA biology, a function intimately linked to high egg quality. Therefore, this study has opened new and critical areas related to meiosis and egg quality that should be explored.

### Supplementary Information

Supplementary Figure 1.

Supplementary Tables.

## Data Availability

The data is available in the manuscript and the associated Supplemental Tables.
