# Supplementary material for: Whole transcriptome screening for novel genes involved in meiosis and fertility in Drosophila melanogaster
Source: Sci Rep. 2024 Feb 13;14:3602. doi: 10.1038/s41598-024-53346-z (PMC10864285; doi:10.1038/s41598-024-53346-z)
Supplement: Supplementary file 1 — Supplementary Figure 1. [file 41598_2024_53346_MOESM1_ESM.pdf]

Supplemental Figures

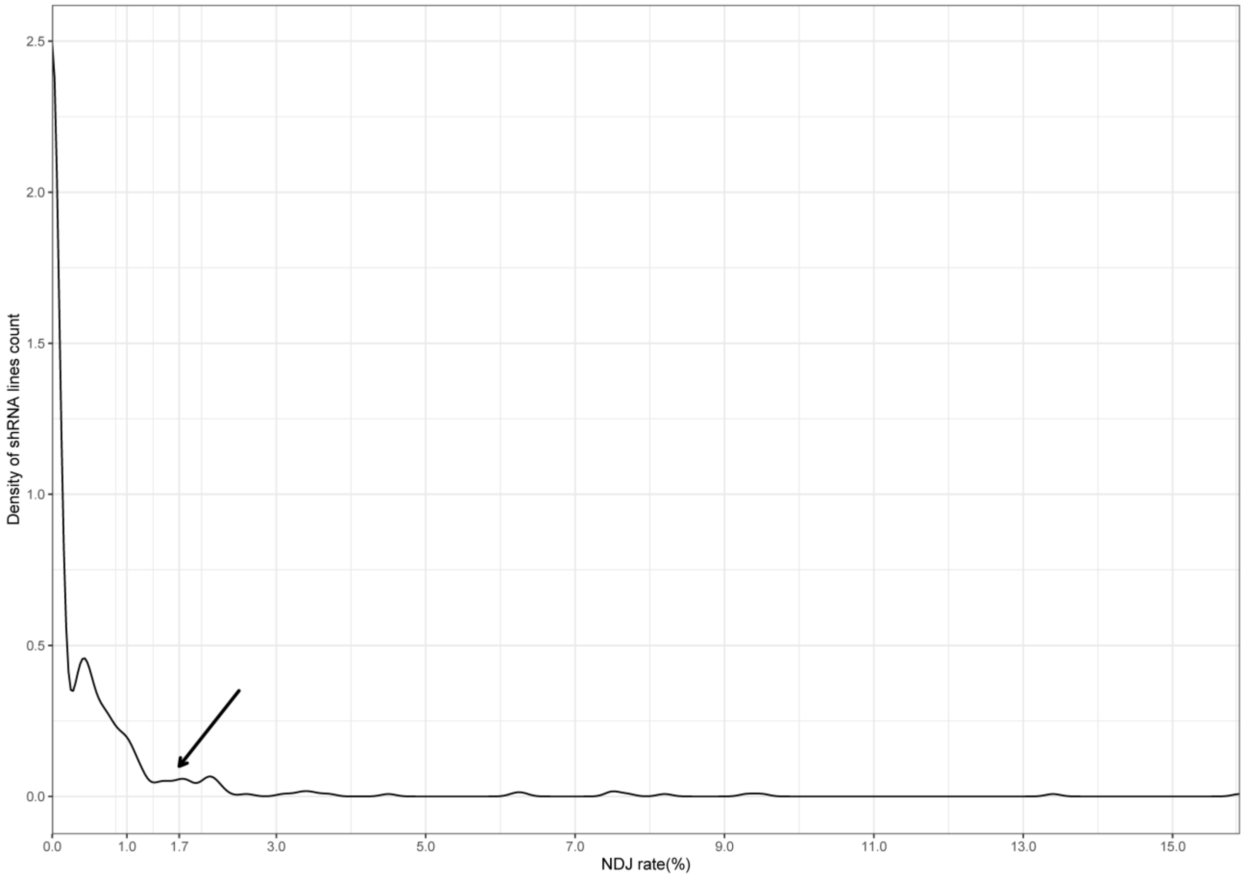

Figure S1: Distribution of RNAi knockdown non-disjunction results. All observed rates of nondisjunction were plotted to determine the frequency of occurrence. Elevated rates of nondisjunction were determined to be about 1.68% based on the distribution of frequencies.
